# Current Trends in Circulating Biomarkers for Melanoma Detection

**DOI:** 10.3389/fmed.2022.873728

**Published:** 2022-04-05

**Authors:** Nancy Huang, Katie J. Lee, Mitchell S. Stark

**Affiliations:** The University of Queensland Diamantina Institute, The University of Queensland, Dermatology Research Centre, Brisbane, QLD, Australia

**Keywords:** melanoma, biomarker, CTC (circulation tumor cells), ctDNA (circulating tumor DNA), miRNA—microRNA, extracellular vesicles (EVs)

## Abstract

Melanomas have increased in global incidence and are the leading cause of skin cancer deaths. Whilst the majority of early-stage, non-metastatic melanomas can be cured with surgical excision alone, ~5% of patients with early melanomas will experience recurrence following a variable disease-free interval and progression to metastatic melanoma and ultimately death. This is likely because of primary tumor heterogeneity and progressive clonal divergency resulting in the growth of more aggressive tumor populations. Liquid biomarkers have the advantage of real-time, non-invasive longitudinal monitoring of tumor burden and heterogeneity over tissue markers. Currently, the only serological marker used in the staging and monitoring of melanoma is serum lactate dehydrogenase, which is not sufficiently specific or sensitive, and is not used routinely in all centers. An ideal melanoma biomarker would be used to identify patients who are at high-risk of primary melanoma, screen for relapse, detect early-stage melanoma, provide treatment outcomes to personalize systemic treatment, follow tumor heterogeneity, provide prognostic data before, during and after treatment, and monitor response to treatment. This review provides a summary of the current research in this field with a specific focus on circulating tumor cells, circulating tumor DNA, microRNA, and extracellular vesicles which may serve to suit these goals.

## Introduction

Melanoma is an aggressive skin cancer with an increased global incidence over recent decades. Melanomas may be diagnosed following a clinical history and skin examination, sometimes with the assistance of total body photography or sequential digital dermatoscopic imaging in higher-risk patients (i.e., previous melanoma, family history, and phenotypic characteristics such as fair skin, high total body nevus count, etc.). The primary tumor is excised for dermatopathological examination, the gold standard for diagnosis and staging (1). If high-risk features are detected (e.g. >1 mm thickness), a sentinel lymph node (SLN) biopsy may be performed to assess for metastatic disease (2, 3).

Staging of melanoma is based on a combination of histological characteristics such as tumor thickness, ulceration, and mitotic rate, and metastasis to lymph nodes and distal sites (4). The American Joint Committee on Cancer TNM staging system is used to determine clinical management and as a guide for prognosis. Overall clinical stage is still a strong determinant of 5-year survival, with high mortality in advanced stages (5). Most melanomas are diagnosed in the early stage (stages I), which can often be completely cured by surgical excision, and thus have 97–99% 5-year survival (6, 7). Unfortunately, some early-stage melanomas recur following excision, and may develop metastases; thus, even melanomas within the same stage can vary in terms of progression and patient survival, likely due to tumor heterogeneity not detected on histopathology (8). SLN-positive individuals have increased treatment options including surgery, advanced therapeutics, and radiological imaging such as CT and PET scans to determine the extent of distal metastatic spread. Regular imaging is common to monitor tumor volume, location, and effectiveness of systemic therapies. This imaging is expensive and not available in all hospital centers.

In Australia, there is a disparity in melanoma incidence and survival between patients living in capital cities and those living in regional and rural areas, with higher incidence and mortality rates in the latter (6, 9). This may be due to a number of factors, such as lower socio-economic status and health literacy, lack of shade in public areas and increased occupational exposure (10). People living in remote and regional centers also have limited access to diagnostic and treatment services, resulting in reduced early detection of thin, easily treatable melanomas.

Systemic therapies for the management of metastatic melanoma (stages III and IV) include targeted therapies such as BRAF and MEK inhibitors, and immunotherapy using check-point inhibitors, including anti-CTLA-4 and anti-PD-1/PD-L1 (11, 12). Response rates to these treatments vary from 22 to 40%, with ~10–15% of patients exhibiting atypical response patterns such as pseudo-progression (12). Patients treated with targeted therapies must have melanomas with *BRAF* V600 mutations, currently determined using tumor biopsies (13).

Consequently, there is a need to identify biomarkers that can detect early-stage melanoma and stratify patients into melanoma recurrence risk groups. This will allow resource prioritization for screening high-risk groups which is particularly important in rural and regional areas, and allow intervention during early-stage disease, shown to be the biggest factor in improving overall survival (OS) (14).

## Circulating Melanoma Biomarkers

A biomarker is a biological molecule that may be detected in bodily fluids such as blood, urine, saliva, or in tissues, and in comparison with a healthy individual or tissue, an increase/decrease of this biomarker is a sign of an abnormal process (15). Circulating biomarkers include tumor cells, nucleic acids (DNA, RNA, microRNA, etc.), extracellular vesicles (EVs), and metabolites, often called a liquid biopsy or a liquid biomarker. Liquid biopsies can have substantial benefits over tissue-based biomarkers as collection is minimally invasive, may allow for longitudinal tracking of a patient's tumor burden, detection of recurrence, and profiling tumor heterogeneity and clonal divergence (16). In addition, liquid biomarkers may be particularly useful when the primary melanoma lesion is limited (e.g. previously excised melanomas) or where tissue sampling may be difficult (13).

Multiple serological protein markers have been explored, with lactate dehydrogenase (LDH) currently the only marker with some clinical value for monitoring treatment response and prognosis; however, its sensitivity and specificity are lacking (3, 4, 17–19). Other markers such as S100B, MIA protein, CRO, PD-L1, IL-8, TIL, osteopontin, and YKL-40 have been explored; however, their clinical utility is limited as many of these markers are associated with other biological processes such as inflammation, infection, autoimmune conditions or other cancers (17, 19). Aside from LDH and S100B, there are no additional clinically validated liquid biomarkers that can be used to detect early-stage melanoma, pre-treatment prognosis, expected treatment outcomes, or monitor treatment outcomes in metastatic melanoma (12, 18).

An ideal biomarker would be sensitive and specific for melanoma to screen high-risk populations and monitor for relapse in early-stage, asymptomatic melanoma patients, and be used to personalize treatments in advanced-stage melanoma (e.g. selecting optimal treatment, identifying when to add adjuvant therapies) to reduce healthcare costs and adverse effects such as toxicity (12, 16). Other favorable qualities would be a test that is low cost, has high throughput capacity, and is readily accessible in rural and regional settings.

## Cellular Profiling

Whole blood analysis is standard practice for melanoma patients to measure blood cell counts. This review will not discuss these analyses, and instead focus on circulating tumor cells.

### Circulating Tumor Cells (CTC)

CTCs are shed from solid tumors into the circulation, and are considered a vital step in the establishment of distant metastases (16, 20). They have a short half-life due to physical and oxidative stress, anoikis, absence of growth factors and cytokines, and cell loss from implantation into capillary beds (16, 21). Detection of CTCs remains challenging despite advances in cell isolation, enrichment, and analysis methods, with detection rates in advanced melanoma patients ranging from 28 to 87% (16, 22). This is due to several factors, including low concentrations in circulation, marker heterogeneity, and lack of standardized method for isolation (20, 22–26).

Patients with early-stage melanoma commonly have only single positive melanoma markers on CTCs, whilst patients with advanced stages not only had higher CTC counts, but also a greater variety of markers (25). Reverse transcription polymerase chain reaction (RT-PCR) is the most commonly used method for detecting CTC markers (sensitivity 1–10 CTCs/mL whole blood, specificity 99.9%); most detection techniques rely on one to two known markers (25, 27–29). Aya-Bonilla et al. compared isolation and detection methods, finding individual detection rates of 28% for multi-marker immunostaining, 42% for a 5-gene panel via RT-PCR, and 53% for a 19-gene droplet digital PCR (ddPCR) assay; however, by combining all three methods, the detection rate was bolstered to 72% (22). Commonly-used CTC markers include tyrosinase, Melan-A/MART-1, MAGE3, MCAM, gp-100, MITF, and *Gal*Nac-T, which have specificities ranging from 85 to 100% but sensitivities from 6 to 95% (Table 1) (24, 27). Other isolation methods include microchips, antibody-coated immunomagnetic beads, and combining RT-PCR with selective fluorescent imaging (26). Therefore, it may be worthwhile adopting a multi-marker approach to improve clinical utility of any one biomarker.

**Table 1:** CTC markers of melanoma.

**Marker(s)**	**Detection method**	**Study group(s)**	**Detection Rate**	**Sensitivity, Specificity, PPV**	**Disease recurrence**	**References**
Tyrosinase	RT-PCR	Stage I–IV (*n* = 212)	Stage I–II: 14% Stage III–IV: 48%	Sensitivity: 22%	88% of relapsed patients had positive markers, 39% of relapsed patients had negative markers over 36 month follow-up period in stage III patients.	(30)
Tyrosinase	RT-PCR	Control (*n* = 52) Stage I–II (*n* = 177) Stage III–IV (*n* = 122)	Control: 0% Stage I–II: 53%^*^ Stage III–IV: 82%^*^	Sensitivity: 65%^*^ Specificity: 100% PPV: 100%	No correlation was noted between relapse and marker positivity.	(28)
MLANA/MART-1	RT-PCR	Control (*n* = 52) Stage I–II (*n* = 120) Stage III–IV (*n* = 91)	Control: 0% Stage I-II: 18%^*^ Stage III–IV: 33%^*^	Sensitivity: 25%^*^ Specificity: 100% PPV: 100%	No correlation was noted between relapse and marker positivity.	(28)
Tyrosinase or MART1	RT-PCR	Control (*n* = 89) Stage I–II (*n* = 236)	*Tyrosinase (initial):* Control: 0% Stage I-II: 5%	*Tyrosinase or MART1 over follow-up period:* Sensitivity: 60% Specificity: 98% PPV: 99%	40% of relapsed patients had positive markers over median 36 month follow-up period.	(31)
			*MART1 (initial):* Control: 2% Stage I-II: 7%			
MLANA ABCB5 TGF-β2 PAX3d MCAM	qRT-PCR	Control (*n* = 152) Stage 0–II (*n* = 154) Stage III-IV (*n* = 76)	*2 or more markers:* Control: 17% Melanoma: 72%	*2 or more markers:* Sensitivity: 71% Specificity: 83% PPV: 86%	MLANA + ABCB5 PPV: 56%, positive in 26% of pts with relapse. 48% of Stage III–IV relapsed patients had MLANA. No correlation for stage 0-II over 45 month follow-up period.	(32)
CD146 antibodies	CellSearch Circulating Melanoma Cell Assay	Control (*n* = 91) Stage III (*n* = 243)	Control: 2% Stage III: 59%	Sensitivity: 59% Specificity: 98% PPV: 99%	48% of relapsed patients had positive markers within 54 month follow-up period (median 17 m)	(24)

* *Figures derived from positivity rates within individual groups provided in the study*.

CTCs have also been detected in early-stage melanoma including melanoma *in-situ* (MIS) (29), with Voit et al. reporting a positive association between detectable CTC counts and progression-free survival (PFS) in stage I-III patients (33). Lucci et al. noted that the detection of one or more melanoma CTCs (per 7.5 mL peripheral blood) in stage III patients was significantly associated with disease relapse within 6 months (24). Fluctuations in CTC counts have been associated with treatment response, disease progression, and OS (16, 29).

New technological advances to enrich and isolate CTCs, and standardization in isolation techniques will likely improve the clinical utility of CTCs to define heterogeneity and occult invasiveness, assess tumor burden, direct treatment decisions, and monitor response to treatment (21, 29).

## Molecular Profiling

Molecular profiling aims to detect changes in signaling and presence of abnormal genetic material that contribute to the development and progression of melanoma. There are numerous other circulation molecules under investigation (including long non-coding RNA), however, this review will focus on circulating tumor DNA, microRNA, and EVs.

### Circulating Tumor DNA

Circulating tumor DNA (ctDNA) are fragments of single or double-stranded DNA released at low concentrations by tumor cells into the circulation, likely by cell apoptosis and necrosis, although the precise mechanism is uncertain (3, 16–18, 34). ctDNA can be measured in plasma or serum, but it has a short half-life as a result of rapid clearance though the kidneys, liver and spleen (16, 34). This timeframe can be extended if with specific collection tubes which preserve the ctDNA at room temperature up to 7 days (35). ctDNA levels are associated with tumor burden, location, and vascularity, and the rate of cellular turnover (16–18). As patients with early-stage or MIS have low tumor burden and vascularity, ctDNA levels are often not detectable in these groups using conventional technology (3, 16, 17). There are also technical issues in detecting and analyzing ctDNA. Firstly, ctDNA only constitutes a small fraction of the total background cell-free DNA (cfDNA), which itself is only present in small quantities in the circulation (16, 34). Ongoing advances in technology, such as the use of ddPCR, are sensitive at detecting low-level ctDNA, and are readily reproducible (36). The proportion of cfDNA also increases with leukocyte lysis, which can occur during blood collection and clotting, reducing the ability to detect ctDNA (16, 37). Consequently, plasma is the preferred source of ctDNA (16). There is however a lack of consistency in blood collection processes, including types of tubes used, time to plasma separation and processing, and storage and transport conditions (16, 37). These differences make analysis and comparison across different study groups troublesome; thus, cut-off values have not been established, reducing the clinical utility of ctDNA (3, 17). Finally, to distinguish ctDNA from cfDNA, specific somatic mutations in the melanoma tumor must first be identified (3). Tumor heterogeneity thereby continues to hinder detection rates, as ~25% of melanoma patients do not have identifiable tumor-derived ctDNA mutations present in the circulation (3, 36).

Despite the challenges, monitoring ctDNA levels in metastatic melanoma patients is non-invasive, with potential clinical utility demonstrated in determining pre-treatment prognosis, monitoring of treatment response, assessment of clonal evolution and minimal residual disease (MRD), and tracking disease progression (16–18, 34). Monitoring MRD is particularly important to confirm complete responders to systemic therapies due to detection limits of radiological imaging (38).

Low or undetectable pre-treatment levels of ctDNA in patients with metastatic melanoma was associated with lower tumor burden and increased PFS and OS, independent of LDH and tumor stage (16–18, 36). However, if detectable levels of ctDNA following surgical excision was detected, this was associated with a poor PFS and OS (34). In patients who had detectable baseline ctDNA, there was a correlation with tumor burden and LDH, suggesting that ctDNA may reveal tumor burden in the absence of imaging (13, 18, 36). In addition, elevated baseline ctDNA levels correlate with reduced overall response rate, and shortened PFS and OS in melanoma patients treated with targeted or immune therapies (16, 17, 34, 39).

There are however limitations to using ctDNA as a predictive biomarker for patients undertaking second-line treatment immunotherapy after failed initial targeted treatment, as there was no association between low ctDNA levels and PFS (39). Additionally, in patients with brain metastases, the levels of ctDNA may be artificially low as there is less ctDNA released into the circulation (40); accurate detection relies on more invasive collection of cerebral spinal fluid (41).

### MicroRNA

MicroRNA (miRNA) are short, non-coding RNA fragments which regulate gene transcription and expression, thereby affecting cell differentiation, proliferation, migration, and apoptosis (3, 16, 42, 43). They are secreted into the circulation, usually in lipid-bound vesicles or in a complex bound by protective proteins and are highly stable even under harsh conditions such as extremes of pH and temperature, multiple freeze-thaw cycles, and long-term storage (3, 14, 16).

Detection of miRNA relies on sequence-matching miRNA molecules (probes, oligos, etc.), and selective and sensitive amplification, but standard techniques such as real-time quantitative RT-PCR can be used. Often low concentrations of miRNA in blood and different expression profiles between serum and plasma samples, and across individuals, can make detection challenging (3, 16), and differences in sample collection and preparation, RNA extraction, normalization methods and storage, limit comparisons between studies (3). Many miRNAs have however been linked to melanoma and metastasis (3).

For example, miR-221 has a role in maintaining cell cycle regulation and proliferation, and so its dysregulation promotes melanoma progression (3, 44, 45). Serum levels of miR-221 were generally undetectable in controls and patients with MIS; however, they were significantly elevated in most patients with stage I melanoma, and associated with increasing clinical stage and tumor thickness (46). Other miRNA regulating cell cycle with altered expression in melanoma include miR-9, miR-150, miR-155, miR-205, and miR-222 (3, 45).

miR-199a-5p promotes melanoma metastasis and angiogenesis, and has been shown to be up-regulated 10-fold in patients with stage III melanoma compared to stage Ia disease (47). Even patients with stage Ib to IIb disease had a 6-fold increase in expression compared to healthy and stage Ia groups (47, 48). Up-regulation of miRNA-199a-5p in conjunction with up-regulated miR-877-3p, 1228-3p, 3613-5p and down-regulated miR-182-5p was associated with higher melanoma stage (stage III or higher) at the time of primary melanoma excision (47). Patients who did not have down-regulated miRNA-182-5p were usually within stages Ib-IIb (47). Additionally, dysregulation of miR-199a-5p, miR-150, miR-15b, miR-33a, and miR-424 increased the risk of melanoma recurrence, with increasing levels associated with tumor burden (16).

These studies are a few examples of dysregulated circulating miRNAs associated with differing melanoma stages relative to healthy controls. When used in isolation, individual miRNAs may not yet be able to be used clinically due to inconsistent expression across patients. The use of a collection or panel of miRNA may result in a more sensitive and specific biomarker (3). Margue et al. (49) showed that miR-301-3p, miR-200c-3p, miR-126-5p, miR-374a-5p, and miR-211-5p, when used in a combination, could differentiate patients with stage I and II melanoma from healthy controls. van Laar et al. also used a combination of 38 miRNAs (MEL38), to differentiate healthy controls from patients who had stage I-IV melanoma (50). We too have identified a panel of seven “melanoma-related” miRNA biomarkers that have prognostic value as a liquid biopsy measured in serum (51). In treatment naive stage IV patients, members of this MELmiR-7 panel were able to detect an increase in tumor burden in 100% of cases and the ability of the panel to define OS proved to be superior to LDH and S100B levels (delta log-likelihood = 11, *p* < 0.001) (51). Levels of circulating miRNA may fluctuate with systemic treatments used to treat late-stage melanoma patients so the use of miRNAs in this context requires further validation.

The use of miRNA as a melanoma biomarker is promising, and whilst several studies focused on an individual miRNA, further research into groups of miRNAs may provide a more sensitive and specific tool to differentiate early-stage melanoma from healthy controls and provide better pre-treatment prognosis.

### Extracellular Vesicles

Extracellular vesicles are membrane-bound particles secreted by all cells, present in all body fluids, and transport cargo (DNA, RNA, miRNA, proteins, lipids, and metabolites) that are representative of the original cell (7, 16, 52, 53). The EVs secreted by tumor cells can deliver tumor-specific molecules to other body sites that may assist tumor survival and proliferation (16). Exosomes, which are nano-sized subset of EVs, released by melanoma cells may also stimulate the migration of endothelial cells, induce angiogenesis to promote distal metastasis, contribute to drug resistance, and may have immunosuppressive actions (54, 55).

Exosomes can be isolated based on physical characteristics such as size, morphology, concentration, cell surface markers or cargo (16, 52, 56, 57). Detection of tumor-derived exosomes with current technologies is difficult, due to their size, low quantities of cargo within each exosome, heterogeneity, and abundance of non-target EVs (7).

In melanoma patients relative to healthy controls, 4–20-fold higher levels of plasma tumor-derived exosomes were detected which persisted despite surgical resection of the primary lesion with no clinical evidence of residual disease (58–60). Exosomes are known to be involved in establishing pre-metastatic niches in cancers including melanoma (61). This persistence of the exosomes, post excision of primary lesions, may be a reason why in some patients, recurrence of metastatic disease occurs many years after initial primary diagnosis. Recently, Wang et al. compared the expression of EV surface markers MCSP, MCAM, CD61, and CD63 in healthy controls and patients who had a history of early-stage melanoma (including MIS) (7). Surface enhanced raman spectroscopy (SERS; an ultra-sensitive detection method) signatures and mapping images showed a distinct separation between the two groups, with higher levels of SERS signals observed in the melanoma patients (7).

## Discussion

The identification and validation of liquid biomarkers has seen significant advancement over the past two decades. The biomarkers discussed in this review all hold great promise to improve early melanoma detection and disease relapse, as well as a prognostic marker at various stages of disease and treatment, and predictive markers to identify patients who would benefit most from systemic therapies, to personalize treatment options (Table 2) (12, 16, 34).

**Table 2:** Strengths and weaknesses of reviewed melanoma biomarkers.

**Biomarker**	**Strengths**	**Weaknesses**
CTC	• Sensitivity may be improved using multi-marker methods • Level fluctuations can be associated with treatment response, disease progression, and OS	• Short half-life in blood • Very low sensitivity in early-stage melanoma • Patients must have known melanoma markers • Levels not correlated with Breslow thickness
ctDNA	• Levels associated with tumor burden • Low or undetectable levels in metastatic melanoma patients associated with increased PFS and OS • May be used to determine first-line systemic therapy	• Short half-life in blood • Often not detectable in early-stage melanoma • Lack of consistent processes for collection, storage and detection • No established cut-off values • Patients must have known melanoma mutations
miRNA	• Highly stable • Multi-marker analysis may increase sensitivity	• Low concentration in blood • Lack of consistent processes for collection, storage and detection
EVs	• Moderately stable • May identify metastatic disease	• Low concentration in blood • Technical challenges with detection • Lack of consistent processes for detection and reporting

CTCs are highly specific melanoma biomarkers as they rely on the detection of known melanoma markers, however this can come as a disadvantage for patients with tumor populations that do not display these markers (25). Whilst some detection methods have reported positive results in patients with early-stage melanoma, overall, the sensitivity remains quite low, likely as a result of more homogenous tumor populations and low circulating concentrations of CTCs in these patients (16, 29, 33). CTCs fluctuate with tumor burden in metastatic melanoma and can thus be used to monitor treatment outcomes and disease progression (16, 29). In metastatic melanoma, CTC counts are also associated with risk of disease recurrence and PFS/OS, and thus may assist in identifying high-risk patients who require more intensive screening and provide prognostic information (16, 24, 29).

ctDNA may have less clinical utility as an early-stage melanoma biomarker, especially in rural and regional settings. This is due to their low sensitivities in early-stage disease, their instability, need for specialized sample tubes, and highly specific processing requirements and storage conditions to prevent sample degradation (3, 16, 17, 37). On the other hand, ctDNA levels can be used to independently predict pre-treatment PFS and OS, personalize treatment options based on expected outcomes, and monitor response to treatment and disease progression (11, 13, 16–18, 34, 36, 39). Detection methods for ctDNA, such as ddPCR, are cost-effective, and next-generation sequencing can allow for high throughput testing (16).

miRNA may be a promising future melanoma biomarker due to its stability, monitoring of clonal divergence, and ability to differentiate healthy controls from patients with early-stage melanoma (3, 14, 16, 46, 51). Certain groups of miRNA markers were able to provide information on risk of recurrence, detect early metastasis, and prognosis (3, 16, 43, 51). RT-PCR is the predominant method used for miRNA detection, which is cost-effective and widely-available, however discrepancies in sequence matching may reduce the sensitivity (3, 16).

Whilst the technical challenges associated with detection of EVs and poorly standardized reporting may limit its use as a melanoma biomarker, steps are being taken to address these issues, and some technologies for detection may allow for high volume testing (7, 16). Multiple EVs are able to differentiate healthy controls from patients with advanced-stage melanoma, and may provide prognostic information (16, 55, 57). The findings from current studies suggest that EVs will be a promising melanoma marker.

## Conclusion

Non-invasive methods such as total body photography for high-risk patients and CT and PET scans for late-stage patients and those suspected of lymph metatasis with enlarged nodes, are available to screen patients, however there are usually cost and significant access barriers present, especially in rural and regional areas (43). On the other hand, obtaining a blood sample to assess for liquid biomarkers is easier, more accessible, and may be more cost-effective and allow for high throughput testing (16, 43). At present, there are currently no clinically-validated liquid biomarkers to distinguish these patients, and thus a melanoma biomarker that is AJCC stage-specific is urgently required.

## Author Contributions

NH and MS conceived the paper and wrote the first draft of the manuscript. NH, KL, and MS contributed to manuscript revision, read, and approved the final submission.

## Funding

MS is supported by the Merchant Charitable Foundation. KL is supported by an NHMRC Dora Lush Basic Research Scholarship. This research was conducted with the support of the NHMRC Centre of Research Excellence in Skin Imaging and Precision Diagnosis (APP2006551) and the ACRF Australian Centre of Excellence in Melanoma Imaging & Diagnosis.

## Conflict of Interest

The authors declare that the research was conducted in the absence of any commercial or financial relationships that could be construed as a potential conflict of interest.

## Publisher's Note

All claims expressed in this article are solely those of the authors and do not necessarily represent those of their affiliated organizations, or those of the publisher, the editors and the reviewers. Any product that may be evaluated in this article, or claim that may be made by its manufacturer, is not guaranteed or endorsed by the publisher.

## Abbreviations

AJCC: American Joint Committee on Cancer
cfDNA: cell-free DNA
CT: computerized tomography
CTC: circulating tumor cells
ctDNA: circulating tumor DNA
ddPCR: digital droplet polymerase chain reaction
EV: extracellular vesicle
LDH: lactate dehydrogenase
miRNA: microRNA
MIS: melanoma *in-situ*
MRD: minimal residual disease
OS: overall survival
PFS: progression-free survival
RT-PCR: reverse transcriptase polymerase chain reaction
PET: positron emission tomography
SERS: surface enhanced raman spectroscopy
SLN: sentinel lymph node
TNM: tumor (T), node (N), and metastasis (M).
